# Active plasmonics in WDM traffic switching applications

**DOI:** 10.1038/srep00652

**Published:** 2012-09-12

**Authors:** Sotirios Papaioannou, Dimitrios Kalavrouziotis, Konstantinos Vyrsokinos, Jean-Claude Weeber, Karim Hassan, Laurent Markey, Alain Dereux, Ashwani Kumar, Sergey I. Bozhevolnyi, Matthias Baus, Tolga Tekin, Dimitrios Apostolopoulos, Hercules Avramopoulos, Nikos Pleros

**Affiliations:** 1 Department of Informatics, Aristotle University of Thessaloniki, 54124 Thessaloniki, Greece; 2 Informatics and Telematics Institute, Center for Research and Technology Hellas, 57001 Thessaloniki, Greece; 3 School of Electrical and Computer Engineering, National Technical University of Athens, 15780 Zografou, Athens, Greece; 4 Institut Carnot de Bourgogne, University of Burgundy, F-21078 Dijon Cedex, France; 5 Faculty of Engineering, Institute of Technology and Innovation, University of Southern Denmark, DK-5230 Odense M, Denmark; 6 AMO GmbH, 52074 Aachen, Germany; 7 Fraunhofer IZM, D-13355 Berlin, Germany

## Abstract

With metal stripes being intrinsic components of plasmonic waveguides, plasmonics provides a “naturally” energy-efficient platform for merging broadband optical links with intelligent electronic processing, instigating a great promise for low-power and small-footprint active functional circuitry. The first active Dielectric-Loaded Surface Plasmon Polariton (DLSPP) thermo-optic (TO) switches with successful performance in single-channel 10 Gb/s data traffic environments have led the inroad towards bringing low-power active plasmonics in practical traffic applications. In this article, we introduce active plasmonics into Wavelength Division Multiplexed (WDM) switching applications, using the smallest TO DLSPP-based Mach-Zehnder interferometric switch reported so far and showing its successful performance in 4×10 Gb/s low-power and fast switching operation. The demonstration of the WDM-enabling characteristics of active plasmonic circuits with an ultra-low power × response time product represents a crucial milestone in the development of active plasmonics towards real telecom and datacom applications, where low-energy and fast TO operation with small-size circuitry is targeted.

The need for higher throughput connectivity with minimal power consumption and size requirements in next-generation High Performance Computing Systems and Data Centers has brought integrated photonics into the spot-light, targeting their penetration into board-level and chip-scale interconnects^1^. In this development, the emerging discipline of plasmonics^2^,^3^ has started to gain ground as the “beyond photonics” chip-scale platform that can enter the interconnect area^4^,^5^,^6^. Plasmonics relies on the propagation of Surface Plasmon Polariton (SPP) modes along metallic stripes yielding in this way strong mode confinements even at sub-wavelength scale^7^, while the underlying metallic circuitry offers a seamless energy-efficient approach to interoperability between light beams and electrical control signals.

The low-energy functional perspective of plasmonics is especially highlighted in the case of Dielectric-Loaded SPP (DLSPP) waveguides that employ a dielectric (e.g. polymer) ridge on top of a metallic stripe. This offers an additional factor for actively controlling electromagnetic wave propagation through the TO^5^,^8^,^9^,^10^,^11^,^12^,^13^ or electro-optic characteristics^14^,^15^ of the dielectric loading. Since the DLSPP mode field reaches its maximum at the metal-dielectric interface, the metallic layer not only supports the plasmonic mode but simultaneously can serve as an energy-efficient heating electrode when electric current is applied to it, exploiting the TO effect. At the same time, fast TO responses are feasible due to the direct contact of the polymer with the underlying metallic film that leads to significantly decreased heat capacity between the waveguide and the electrode, yielding immediate change of the effective index of the propagating SPP mode when the TO effect occurs. DLSPP waveguides are, to the best of our knowledge, the only plasmonic waveguides that have been already employed for active manipulation of SPP signals in a number of functional circuitry implementations like TO modulation^10^,^11^, On/Off gating^9^, switching^12^,^13^ and power monitoring^16^, greatly benefiting also from their recent successful interfacing with the low-loss silicon photonic waveguide transmission platform^5^,^8^,^17^,^18^. The progress made so far on active DLSPP-based circuits suggests the TO effect as the most mature mechanism that can be exploited currently towards bringing low-energy active plasmonics in true data traffic environments: TO tuning of Polymethylmethacrylate (PMMA)-loaded SPP -based racetrack resonators has been recently demonstrated by the authors requiring only 3.3 mW of power^8^, providing the first solid proof of the low power characteristics of the DLSPP platform. In addition, we have recently presented an active PMMA-loaded SPP-based TO switch with 90 *μ*m plasmonic phase arms operating with single-channel 10 Gb/s optical data traffic^9^, successfully evaluating for the first time the performance of active DLSPPs in true data traffic environments.

However, the transition to practical active plasmonic circuits can only be completed by proving their credentials in true WDM applications in order to support the complete broadband portfolio of optics. The dimension of WDM appears currently as the mainstream approach in photonic Network-on-Chip (NoC) implementations towards increasing the aggregate on-chip throughput; Silicon-on-Insulator (SOI)-based TO switching elements employed so far in NoC deployments^19^,^20^ have adapted their design to the WDM-enabling framework, aiming to deliver both low-power and fast reconfigurable dynamic link characteristics^21^. In addition, the case for using TO plasmonics instead of the low-loss and more mature silicon photonics platform is still lacking a solid evidence, since TO SOI-based photonic switch circuitry can offer power and switching time values that go down to mW and *μ*s scales, respectively^22^,^23^,^24^,^25^,^26^. In the present communication, we report on the smallest TO PMMA-based DLSPP switch relying on an Asymmetric Mach-Zehnder interferometric (A-MZI) configuration with only 60*μ*m long active phase branches and performing error-free with 4 data carrying channels in dynamic conditions, with each channel being modulated with 10 Gb/s data. The maximum power penalty was measured to be 3.6 dB for a Bit Error Rate (BER) of 10^−9^, with the power consumption being only 13.1 mW and the response time found to be 3.8 *μ*s. The obtained power consumption and response time values render the DLSPP technology as a powerful TO switch platform in applications where fast, low energy and low footprint TO switching circuitry is needed. They lead to a power × response time product that is lower compared to undoped TO SOI-based^22^,^23^,^24^,^25^,^26^ or polymer-based^27^,^28^ switches reported so far, providing the first tangible advantage of active TO plasmonics over the well-established silicon nanophotonic waveguide platform in WDM data traffic environments.

## Results

### A-MZI layout and principle of operation

The DLSPP waveguide operation relies on the SPP field confinement by a dielectric ridge placed on top of a metal stripe supporting the SPP propagation. The DLSPP waveguide characteristics, i.e., the mode field confinement, effective index and propagation length, are thereby strongly influenced by the width and height of the employed dielectric ridge^29^. Considering PMMA ridges placed on gold stripes to be operated at telecom wavelengths (~1.55 *µ*m), the optimum ridge dimensions, ensuring tight mode confinement (<1 *µ*m) and relatively long propagation (~50 *µ*m) of DLSPP modes, were found to be about 500 nm and 600 nm for the ridge width and height, respectively^29^. In this configuration, the DLSPP mode fills practically the whole PMMA ridge, so that its heating by an underlying gold stripe (via transmitting through it signal currents) modifies very efficiently the DLSPP mode effective index via changing the PMMA refractive index^10^,^11^,^12^,^30^. In fact, theoretical estimations^10^ and calculations^30^ have shown that these ridge dimensions promise TO modulation and switching with record-low driving powers (~1 mW) and response times (~1 *µ*s). Moreover, a temperature increase by ~61 K in PMMA requires an interaction length of ~120 *μ*m for π phase shift at telecom wavelengths that would induce excessive propagation losses^10^. These losses can be greatly reduced by considering only 60 *μ*m interaction length, equivalent to typical propagation length of radiation in DLSPP waveguides, achieving in this way a phase shift of π/2. The remaining needed π/2 phase shift can be completed by making use of an A-MZI configuration, in which this phase shift is permanently introduced in one of the MZI arms by slightly widening the PMMA ridge over a short distance. The width dispersion curves for the DLSPP mode effective index^29^,^30^ indicate that an increase of the 600-nm-high PMMA ridge width from 500nm to 700 nm would result in an increase of the DLSPP mode effective index by ~0.06, implying that the length of 6 *µ*m of a widened ridge section should be sufficient to introduce a π/2 phase shift.

**Figure 1:**
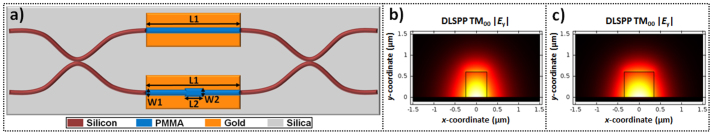
Asymmetric Mach-Zehnder interferometer. (a) Schematic layout. The lower plasmonic branch is widened in order to introduce a default asymmetry, (b) fundamental quasi-TM mode of the 500×600 nm^2^ PMMA-loaded SPP waveguide, (c) fundamental quasi-TM mode of the 700×600 nm^2^ PMMA-loaded SPP waveguide.

Fig. 1(a) illustrates the employed A-MZI, comprising two DLSPP waveguides as its active, electrically controlled branches that are incorporated between two silicon coupler stages. The upper plasmonic arm of the A-MZI has a length of L1 = 60  *μ*m and a PMMA ridge cross-section with W1 = 500 nm width and 600 nm height. These dimensions ensure the existence of a single TM mode inside the polymer at telecom wavelengths, as depicted in Fig. 1(b). The lower plasmonic arm has again a total length of 60 *μ*m and identical PMMA ridge dimensions, employing however a L2 = 6 *μ*m long DLSPP waveguide section that is widened from W1 to W2 = 700 nm. This widened DLSPP waveguide section introduces a default phase asymmetry of π/2 between the two MZI arms, taking advantage of the higher effective refractive index value experienced by the DLSPP waveguide mode in the PMMA ridge. This larger width has been carefully chosen in order to keep the lower branch mono-mode and avoid any interference between fundamental and higher order modes. The field map of this mode in the widened waveguide is shown in Fig. 1(c).

The switching operation in all-plasmonic MZIs has already been described^30^,^31^,^32^ and relies on inducing a total phase shift of π between their two arms. However, in our A-MZI, only a π/2 phase shift is required for switching operation due to the default asymmetry. Here, the phase shift is obtained by changing the temperature locally in one or two MZI arms as a result of the injected electric current. This TO effect has already been predicted^30^ and observed^10^,^12^ on similar devices, and is now well understood. The effective index of the mode that propagates on one arm is modified by the heat compared to the second arm, inducing a phase difference at the output that ideally is π. By electrically controlling the upper A-MZI arm, a negative phase shift is experienced by the propagating DLSPP waveguide mode as a result of the negative thermo-optic coefficient (TOC) of the PMMA that equals −1.05×10^−4^ K^−1^. When the induced phase shift equals π/2, the phase difference between the modes travelling through the two MZI branches equals π and therefore the whole mode power is exported to the BAR output of the device. On the contrary, when the same current level applies only to the lower MZI plasmonic branch, the default MZI phase asymmetry is cancelled out due to the –π/2 thermo-optically induced phase shift and, thus, the whole mode power emerges at the CROSS port of the MZI.

As such, complete On/Off switching operation can be obtained by electrically driving only the upper plasmonic arm of the switch when On operation is targeted, while the electric current has to flow only through the lower plasmonic arm during Off operation. However, it should be noted that high-quality switching can be obtained even if only one of the two plasmonic MZI branches is thermo-optically addressed. The initial π/2 phase asymmetry results in biasing of the MZI at its quadrature point shifting its operational state to the linear regime of its output power transfer function when no electrical driving signal is present. This asymmetry provides thereby the possibility for high performance switching that requires only a (thermo-optically induced) π/2 instead of π phase shift, offering in this way a simple and passive mechanism for reducing the required energy level and the active plasmonic arm length for a given maximum service temperature. The reduction in the PMMA-loaded plasmonic arm length is highly beneficial towards bringing down the device losses, since the limited maximum service temperature of the PMMA necessitates a DLSPP waveguide length of over 100 *μ*m^10^ in case a full π-phase shift has to be thermo-optically induced, leading to total plasmonic branch propagation losses of close to 10 dB. In the case of the 60 *μ*m-long DLSPP-based A-MZI that we present, the total propagation losses of the plasmonic part are reduced down to 6 dB, yielding a total loss value including the Si-to-DLSPP and DLSPP-to-Si junctions of close to ~11 dB.

### Device characterization and single-channel switching experiment

**Figure 2:**
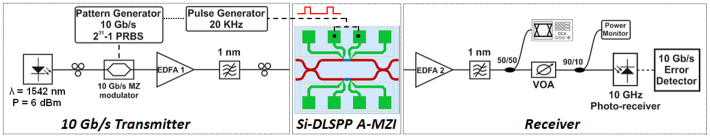
Single-channel experimental setup. The experimental setup comprises a 10 Gb/s single-channel transmitter, a hybrid Si-DLSPP A-MZI and a receiver. Amplifiers are employed in both transmitting and receiving stages. The A-MZI is electrically controlled by a 20 KHz electrical clock pulse train that is injected into its upper branch.

Fig. 2 depicts the experimental setup that was used for obtaining the static TO transfer function of the DLSPP-based A-MZI and for evaluating its performance in single-channel 10 Gb/s data traffic conditions. A Continuous Wave (CW) laser beam at 1542 nm was modulated into a 10 Gb/s optical Non-Return-to-Zero (NRZ) data signal by a Mach-Zehnder modulator (MZM). Subsequently, the modulated signal was amplified by a high-power Erbium Doped Fiber Amplifier (EDFA), providing 30 dBm output power, filtered and launched into an electrically controlled SOI-DLSPP A-MZI. The output of the A-MZI was amplified by EDFA2 providing 10 dBm output power, filtered and detected by a photo-receiver and an error detector.

**Figure 3:**
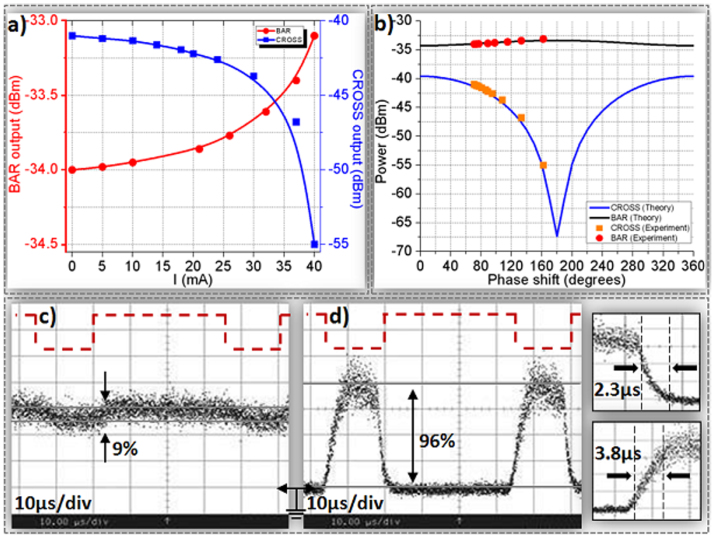
CW injection. (a) Static TO transfer functions for the CROSS and BAR output ports of the A-MZI, (b) theoretically calculated transfer function for the CROSS and BAR output ports of a symmetric MZI showing the region confirmed by the experimentally obtained transfer function of the A-MZI, (c) BAR output TO modulation, (d) CROSS output TO modulation and rise/fall times (insets). Dashed red lines in (c) and (d) show the corresponding electrical control signal.

The static output power versus injected electric current transfer function of the A-MZI is shown in Fig. 3(a) and was obtained through bypassing the Mach-Zehnder modulation stage and launching the 1542 nm CW beam directly in the A-MZI, applying at the same time a Direct Current (DC) at the upper A-MZI branch. As can be noticed, the power level at the CROSS port of the switch decreases with increasing current values, revealing an extinction ratio of 14 dB at 40 mA. The power level at the BAR port increases with electric current, reaching however a poor extinction ratio of only 0.9 dB at a 40 mA current level. The poor extinction ratio value was due to the 95:5 coupling ratio of the silicon directional couplers employed at the MZI's input and output ports, which resulted by an unfortunate design error and prevented the realization of a high performance 2×2 switch. Based on the theoretically expected output power transfer function of a symmetric MZI equipped with 95:5 Si couplers, the experimentally obtained static A-MZI transfer functions for both its CROSS and BAR ports reveal an initial phase biasing point of the A-MZI at approximately 70°, close to the originally intended π/2 value. This is shown in Fig. 3(b), where the theoretically calculated transfer functions for the CROSS and BAR ports, respectively, of a symmetric MZI with 95:5 in- and out-coupling stages versus the injected phase shift are depicted and an almost perfect fit of the corresponding experimentally obtained curves for the 60 *μ*m-long DLSPP-based A-MZI is achieved when considering a 70° phase offset. The injection of 40 mA DC current to the MZI's upper branch caused a thermo-optically induced phase shift of ~−90°, leading to a total phase difference of approximately 160° between the two CW constituents propagating through the different MZI arms. The phase shift of −90° translates into a ~61 K temperature change of the upper MZI plasmonic arm by using the well-known formula 
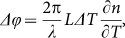
 where *Δϕ* is the thermo-optically induced phase shift, *λ* is the wavelength, *ΔΤ* is the temperature change, *L* is the heated waveguide length and 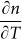 is the PMMA's TOC. The resistance of the 60 *μ*m-long plasmonic A-MZI branch was found to be 6.8Ω at 0 mA current level, rising to 8.2Ω for a DC current of 40 mA due to the temperature-dependent gold resistivity. So, the consumed power for 40 mA driving current was ~13.1 mW.

The dynamic response of the A-MZI switch was evaluated by applying electrical rectangular pulses (noted with red solid line in Fig. 2) of 35 *μ*s duration at a repetition rate of 20 KHz and a nominal electric current peak value of 40 mA at the upper MZI arm. Fig. 3(c)–(d) illustrate the CW traces emerging at the BAR and CROSS ports of the device, revealing a reduction at the CROSS port power when an electrical control pulse is present. The modulation depth of the BAR and CROSS port output signals was 9% and close to 96%, respectively. The rise and fall time values were measured at the CROSS output port and were found to be 3.8 *μ*s and 2.3 *μ*s, respectively, as shown in the corresponding insets. The 3.8 *μ*s rise time relates to the time required by the heated plasmonic waveguide to cool down to its initial steady-state room temperature, while the 2.3 *μ*s fall time is associated with the time required for heating the plasmonic waveguide.

**Figure 4:**
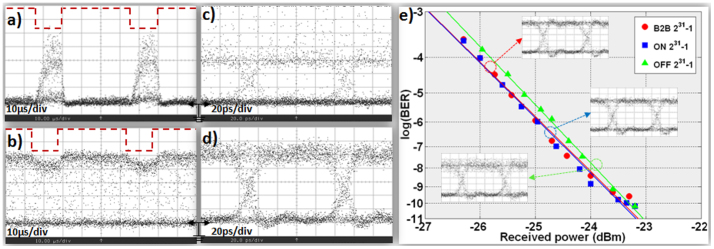
Data injection. Modulation with 35 *μ*s electrical rectangular pulses at 20 KHz repetition rate for 10 Gb/s (a) data trace at the CROSS port, (b) data trace at the BAR port, (c) eye diagram at the CROSS port, (d) eye diagram at the BAR port. (e) (2^31^-1) BER curves for a single channel at B2B, ON and OFF states.

Single-channel data switching was obtained by modulating the 1542 nm CW data signal with a 10 Gb/s 2^31^-1 NRZ data sequence and launching this data stream into the DLSPP-based A-MZI with its upper plasmonic arm being controlled by a 20 KHz electrical clock pulse train with 35 *μ*s pulse duration. Fig. 4(a)–(b) illustrate the data pulse traces observed for the CROSS and BAR ports of the A-MZI, respectively, with the red dashed line showing the corresponding electrical control driving signal. The corresponding eye diagrams are depicted in Fig. 4(c)–(d) for the CROSS and BAR output signals, respectively. As can be noticed, inverted mode operation is obtained at the CROSS port with an extinction ratio of close to 14 dB. The BAR port exiting signal follows the electrical control pattern showing, however, a poor extinction ratio of only 0.9 dB. The noise level of the optical data pulses emerging at the CROSS port resulted to an amplitude modulation of close to 3 dB and was due to the EDFA2 output noise, since the optical power exiting the A-MZI at its CROSS port and entering the EDFA2 was below the amplifier's input power requirements. Fig. 4(e) illustrates the BER measurement curves obtained for the Back-to-Back (B2B) as well as for the ON and OFF states of the switch. The OFF state was determined by the complete absence of any electrical control signal, so that the data signal emerges at the CROSS port, which was also used for monitoring the BER performance. During ON state, the A-MZI was driven by a constant DC current level of 40 mA, enforcing the optical data to emerge at the BAR port, which was used in this case for BER measurement purposes. Error-free performance was obtained in both cases, with the ON state BER curve revealing an almost 0 dB power penalty while a negligible and close to the statistical error power penalty of 0.15 dB was obtained during the OFF state operation with respect to the B2B curve. The corresponding eye diagrams for B2B and for ON and OFF operational states are included as insets in the same figure.

It should be mentioned that our A-MZI device did not allow for the wire-bonding of both MZI arms, so that its performance could not be evaluated by applying electrical current to both MZI branches alternatively. However, its operation with two complementary electrical control signals applied at the two MZI branches would not affect its peak power requirements, since the maximum phase shift needed also in that case will be again π/2. The use of two control signals would only improve the extinction ratio performance, since this would allow for complete π-phase difference between the CW constituents, without increasing the necessary amount of power.

### WDM switching experiment

**Figure 5:**
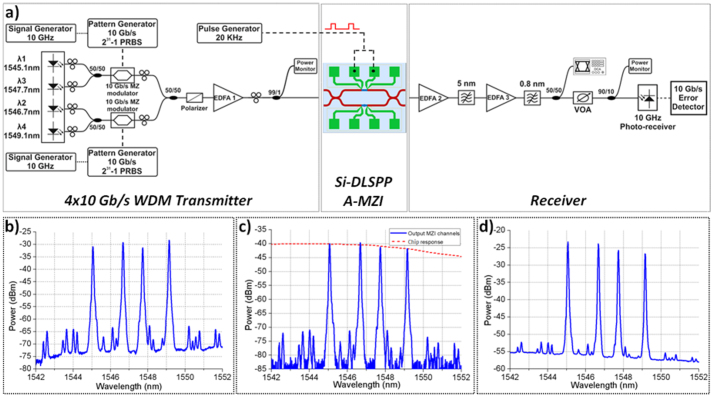
WDM switching experiment. (a) Experimental setup and the 4-channel spectrum at (b) MZI input, (c) directly at the MZI output before entering EDFA2, (d) after the receiver's pre-amplification stage. The spectral response of the chip including the A-MZI and the TM grating couplers is shown with the red dashed line in (c).

The experimental setup used for the WDM switching application is shown in Fig. 5(a). Four CW laser sources emitting light at 1545.1 nm, 1546.7 nm, 1547.7 nm and 1549.1 nm were multiplexed in pairs and each channel pair was modulated to a 10 Gb/s 2^31^-1 NRZ data signal by a corresponding Ti:LiNbO_3_ MZM. The four data streams were multiplexed into a 4×10 Gb/s WDM signal that was then amplified by a high-power EDFA providing 31 dBm power at the input of the electrically controlled DLSPP-based A-MZI. After exiting the A-MZI, the WDM data signal was amplified in a two stage EDFA with a 5 nm optical bandpass filter (OBPF) residing between the two stages for out-of-band Amplified Spontaneous Emission (ASE) noise rejection. Individual channel isolation was performed after EDFA3 in a 0.8 nm OBPF and the signal quality was evaluated by means of a sampling oscilloscope and a photo-receiver followed by an error-detector.

Fig. 5(b) illustrates the 4-channel spectrum after being amplified by EDFA1 and prior entering the A-MZI. The unequal power profile of the WDM signal owes mainly to the respective non-flattened gain profile of the high-power EDFA1 as well as to the energy transfer to the four-wave mixing (FWM) terms that originate as a result of the high-power signal propagating in the fiber link between EDFA1 and the A-MZI. The FWM components within the 1545–1549 nm wavelength window are, however, out-of-band with respect to the data channels so that they can be easily isolated by subsequent filtering stages. Fig. 5(c) shows the corresponding 4-channel spectrum directly at the output of the A-MZI, revealing that their spectral power profile has been altered compared to the corresponding A-MZI input profile and follows the power distribution dictated by the A-MZI's spectral response including the TM grating couplers, also depicted in this figure by the dashed line. As can be noticed, the spectral response of the chip over the wavelength window of interest has a clear wavelength-dependent behavior with the transmission losses increasing by 3 dB when moving from 1545 nm to 1550 nm, originating from the spectral response of the TM grating coupler stages that had a resonance dip around 1560 nm. The multi-wavelength signal after exiting the receiver's pre-amplification stage (EDFA2) is depicted in Fig. 5(d). The wavelength-dependent gain profile of EDFA2 promoted the amplification of shorter wavelengths being closer to the peak spectral gain of the amplifier, leading in this way to different power levels and also different optical signal-to-noise ratios (OSNR) between the four received channels.

**Figure 6:**
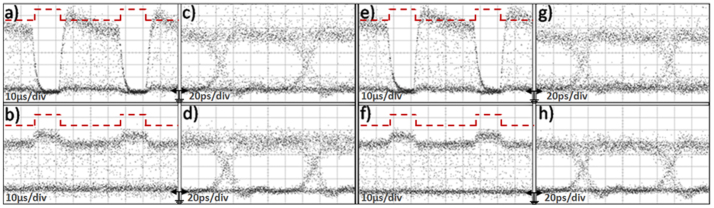
Data injection. Modulation with 15*μ*s electrical rectangular pulses at 20 KHz repetition rate for 10 Gb/s (a) data trace at the CROSS port (channel 1), (b) data trace at the BAR port (channel 1), (c) eye diagram at the CROSS port (channel 1), (d) eye diagram at the BAR port (channel 1), (e) data trace at the CROSS port (channel 2), (f) data trace at the BAR port (channel 2), (g) eye diagram at the CROSS port (channel 2), (h) eye diagram at the BAR port (channel 2).

Fig. 6 illustrates representative data traces and eye diagrams for channel 1 (λ1) and channel 2 (λ2) signals recorded at the A-MZI CROSS and BAR output ports. The electrical control signal shown with the dashed curves had a repetition rate of 20 KHz, with rectangular pulses of 15 *μ*s duration and an electric current peak value of 40 mA. Successful operation of the device was obtained revealing again inverted mode operation at the CROSS port with an ER value close to 14 dB. The BAR output port had again a poor ER performance, not exceeding 0.9 dB, due to the 95:5 coupler splitting ratio. The non-perfectly rectangular shape of the CROSS-port output data packets that yields an amplitude modulation of close to 1 dB originates from the noise accumulated when being amplified in the receiver's stage amplifier units (EDFA2 and EDFA3), since the signal power emerging at the CROSS port was again below the input power range requirements of the EDFA2 pre-amplifier. Similar results for both CROSS and BAR output ports were also obtained for data channels 3 and 4 at λ3 and λ4 wavelengths, respectively (see Supplementary Fig. S1).

**Figure 7:**
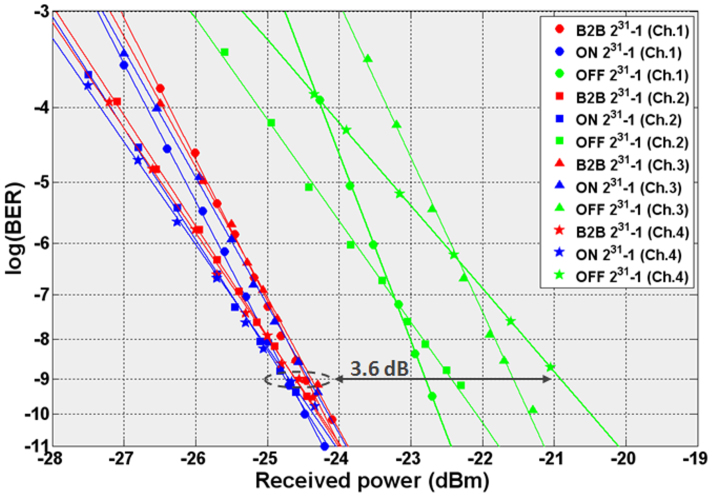
WDM switching BER measurements. BER curves for all channels for B2B and during ON and OFF operational states.

Fig. 7 presents the BER curves obtained for the four channels for the B2B case and when the A-MZI operates in both ON and OFF operational states. Since the intention has been to monitor the signal degradation of the A-MZI device and not of the complete chip including its lossy in- and output grating coupler stages, a straight silicon waveguide employed on the chip has been used for the B2B case. During ON operation, the BER curve was recorded at the BAR output port controlling the A-MZI with a DC electric current value of 40 mA. When operating in OFF state, the BER curve was obtained for the CROSS output port. In both ON and OFF operational states error-free performance was monitored. The ON state shows a negative power penalty close to the statistical error of 0.2 dB for all four channels compared to the B2B curve. When operating at OFF state, the power penalties were ranging between 1.7 dB and 3.6 dB measured at a BER value of 10^−9^ for the four channels. The enhanced power penalty values compared to the corresponding ON state performance owes mainly to the 8 dB lower power level received at MZI's CROSS output (−26 dBm) against its BAR port during ON state (−18 dBm). The −26 dBm of power entering EDFA2 were below the required input power of the amplifier yielding in this way excess noise levels that affected the BER performance at the receiver. Moreover, the power penalty increases with the channel wavelength, being the result of the wavelength-dependent gain and OSNR profile experienced by the four channels during amplification in EDFA2. Finally, the different slopes observed between the BER graphs of channels 1, 3 and the BER curves of channels 2 and 4 owe to the use of a separate data modulation stage per channel pair at the 4×10 Gb/s transmitter end.

## Discussion

The successful demonstration of WDM switching using a DLSPP-based A-MZI device constitutes a major step towards bringing active plasmonics into the realm of true datacom and telecom traffic applications, since the progress made so far in the area of active plasmonics is mainly limited to proof-of-principle experimental demonstrations^8^,^10^,^11^,^12^,^13^,^15^,^33^,^34^,^35^ that cannot certainly be considered as being close to real datacom testing procedures. Even in the area of purely passive plasmonic waveguides, their WDM signal integrity and data carrying credentials were only recently addressed^36^. This step is, however, crucial in order to allow their performance evaluation in real network conditions and address their advantages and drawbacks when it comes to practical application-driven technology developments. All the work carried out so far on active plasmonics has indicated a remarkable potential for low-footprint, low-energy and eventually ultra-fast circuitry, but the transition from a highly promising to a tangible and practical technology has inevitably to proceed along their employment in practical WDM-enabling functional elements.

The WDM DLSPP-based A-MZI switch presented in this work reveals also a clear advantage compared to the Silicon-on-Insulator and the polymer-based TO waveguide technology platforms, as it provides the lowest, to the best of our knowledge, power consumption × response time product over every type of undoped thermo-optically addressed nanophotonic MZI switch. This product, which actually provides also the approximate amount of energy that is dissipated when changing the switching state, has been commonly used for comparing TO switching elements in terms of their utilization in applications where both low energy and fast operation are required^27^.

Table 1 provides an overview of the respective performance metrics reported for different TO MZI switch implementations relying on SOI-based and polymer-based nanophotonic waveguides, which exhibit either the smallest power-time product or the lowest energy consumption or the lowest response time among all TO MZI switches demonstrated so far. TO switches exploiting doped silicon nanophotonic waveguides for using the waveguide core itself as the resistive heater^37^ have been excluded from this comparison, since the aim has been to compare the TO efficiency properties of waveguide technology platforms without assuming any modifications in their intrinsic waveguide characteristics in favor of improving their thermo-optical addressing properties. In addition, only single-control driving schemes have been considered in order to evaluate the response times of the waveguide material platform without taking into account any architectural intervention in the form of differential driving techniques^37^, which are well-known for their switching time reduction advantages. This Table shows that the DLSPP-loaded A-MZI TO switch offers the lowest power-time product using also the shortest active region length, rendering it suitable for TO switching applications where both low energy and low response times are required. The low power operation comes both from its asymmetric arrangement as well as from the inherent DLSPP waveguide characteristics: the asymmetric arrangement allows complete switching operation requiring only a π/2 thermo-optically induced phase shift, reducing in this way the required energy compared to switch architectures where the complete π-phase difference has to originate from the TO effect. At the same time, the DLSPP waveguide geometry includes a metal stripe as a part of its configuration (ensuring the very existence of SPP modes), resulting in the metallic film being directly attached to the dielectric loading and, more specifically, at the site of the waveguide mode intensity maximum. This allows for efficiently transferring the heat to the dielectric loading reducing power consumption, while it also takes advantage of fast thermal diffusion in metals facilitating low switching times. SOI-based TO waveguides can hardly be improved simultaneously in both power consumption and time response, since separate elements are usually used as heaters located at a certain distance from a silicon waveguide core. Sub-mW switching can be achieved by removing the waveguide substrate and using free-standing waveguides for increased heating efficiencies^23^,^24^, which are however achieved at the expense of increased response times. The smallest response time among the single-driving SOI-based TO switches has been achieved by using integrated NiSi waveguide heaters, while featuring a power consumption of 20 mW^22^. On the other hand, polymer-based switches can lead to very low energy consumption values^27^,^28^ suffering however from high response time requirements.

The advantages of the DLSPP technology are put in a better perspective when taking into account the different maturity levels of the considered TO waveguide platforms, since both polymer-based and (even more so) sub-micrometer SOI-based waveguide technologies are several steps ahead with respect to the DLSPP maturity level. The DLSPP-based A-MZI switch is the first active plasmonic device performing with WDM traffic, suggesting that there is still plenty of room available for optimizing its performance, i.e., power, response time and footprint characteristics. The performance can easily be improved by applying perfect 3-dB coupling stages instead of the 95:5 couplers employed in this device, which would then result in extinction ratio values higher than 20 dB for both switch output ports. Moreover, the roadmap towards reducing power consumption, footprint and losses has been already identified in the recent demonstration of DLSPP TO tuning elements that use Cycloaliphatic Acrylate polymer as dielectric loading instead of the PMMA^12^,^13^. The Cycloaliphatic Acrylate polymer has a three times higher TOC compared to PMMA, implying that its use as the dielectric loading in the 60 *μ*m long active DLSPP waveguide arms of our A-MZI switch would lead to approximately three times lower power consumption requirements, bringing the power envelope closer to the sub-mW regime as expected from theory^30^. Alternatively, this scheme could be used for reducing the footprint and as such the losses of the A-MZI device. A driving current of 40 mA as used in our WDM switching experiments would need only 20 *μ*m long active regions instead of 60 *μ*m, decreasing plasmonic propagation losses by 4 dB and leading to the smallest ever reported TO switch. Finally, while noting that the thermal management in DLSPP waveguide components has yet to be optimized, one should also bear in mind that, at any rate, response time reduction could be realized by applying well-known differential driving schemes^37^.

In conclusion, we have demonstrated the first active plasmonic device operating with true WDM traffic. An A-MZI switching structure employing silicon-based coupler stages and TO PMMA-based DLSPP waveguides as its active arms has been shown to provide error-free switching functionality with 4×10 Gb/s incoming data traffic, requiring only 13.1 mW of power and having on/off response times of 3.8 *μ*s and 2.3 *μ*s, respectively. These results verify the potential of plasmonics in developing fast and low-power TO switches with small footprints, providing the smallest power-time product among all undoped SOI- and polymer-based nanophotonic TO switching structures. This clear advantage of the DLSPP TO switch technology platform over respective SOI- and polymer-based devices can pave the way towards a whole new class of ultra-small, energy efficient and fast TO routing fabrics relying on hybrid silicon-plasmonic waveguide platforms, where low-loss silicon is used for the passive functions and DLSPP waveguide components are exploited for the active circuit parts^5^.

## Methods

### SOI-DLSPP A-MZI and waveguide platform fabrication

The SOI motherboard relied on a 400×340 nm^2^ silicon rib waveguide platform with a 50-nm-thick remaining slab to support TM light propagation and was equipped with TM grating couplers as input/output interfaces. Silicon coupler stages were employed for the A-MZI input/output ports. The plasmonic (DLSPP) parts of the A-MZI were hetero-integrated on the SOI rib waveguide platform. The hosting area of the DLSPPWs on the SOI motherboard was etched forming a 200-nm-deep recess in the buried oxide (BOX) of the SOI substrate. The DLSPPWs inside the recess consisted of 500×600 nm^2^ PMMA-made strips placed on top of 3- *μ*m-wide and 65-nm-thick gold films. The interface between the silicon and plasmonic parts of the hybrid A-MZI was attained through a butt-coupling approach following the same design specifications described in^8^. The length of the A-MZI active plasmonic arms was 60 *μ*m and its asymmetric biasing was obtained by using a 700-nm-wide and 6- *μ*m-long DLSPPW section at its lower branch. The total fiber-to-fiber losses of the A-MZI were 40 dB while the insertion losses of only the A-MZI were 11 dB. The high fiber-to-fiber loss value includes 26 dB of losses that were introduced by the two TM grating coupler stages (each grating coupler had an insertion loss of 13 dB) and 3 dB losses owing to propagation in the silicon parts between the TM grating coupler stages and the A-MZI input/output ports. The 11 dB A-MZI device losses are analyzed in 5 dB coming from the Si-to-DLSPP coupling interfaces (2.5 dB per coupling interface) and 6 dB that were the plasmonic propagation losses in the 60 *μ*m long plasmonic sections.

### Single-channel experimental setup

A CW signal at 1542 nm was fed into a Ti:LiNbO_3_ MZM driven by a 10 Gb/s MP1763C Anritsu pattern generator and was consequently modulated to a 10 Gb/s 2^31^-1 NRZ data sequence. Subsequently, a high-power EDFA (EDFA1) was utilized to attain adequate output power of 30 dBm for the modulated signal before entering the A-MZI. Electrical rectangular pulses at 20 KHz repetition frequency were provided by a SRS DG535 digital delay/pulse generator to the metal pads of the DLSPPW in order to control the A-MZI in a dynamic way. The corresponding static TO transfer function measurements were obtained by using a DC current instead of the electrical pulses. After propagation through the A-MZI, the output signal was amplified by an EDFA with 10 dBm output power for an input power of −15 dBm (EDFA2) and was filtered in a narrow OBPF with 1 nm 3-dB bandwidth. The data signal was then split and simultaneously detected by a 30 GHz bandwidth HP 83480 A sampling oscilloscope and a 10 GHz 3-dB bandwidth DSC-R402PIN photo-receiver followed by a 10 Gb/s MP1764C Anritsu error detector for BER measurements. A 20 GHz MG3692B Anritsu signal generator was employed so as to generate the 10 GHz reference signal for the transmitter and the receiver.

### Multi-channel (WDM) experimental setup

Four distributed feedback (DFB) lasers emitting CW light at 1545.1 nm, 1546.7 nm, 1547.7 nm and 1549.1 nm, respectively, were used as the four transmitter channels in the WDM switching experimental setup. The four CW signals were initially multiplexed using fiber 3-dB couplers and modulated in pairs (channels 1 and 3 together and channels 2 and 4 forming another pair) by two Ti:LiNbO_3_ MZMs driven by 10 Gb/s data and data bar outputs of the MP1763C Anritsu pattern generator. In this way, four 10 Gb/s 2^31^-1 NRZ data sequences were generated with channels 1–3 and channels 2–4 carrying decorrelated signals. All four channels were subsequently combined into the same optical link via a fiber 3-dB coupler. The multi-wavelength data signal was then amplified by a high-power EDFA (EDFA1) providing 31 dBm output power before being fed into the SOI-DLSPP A-MZI. A polarizer in conjunction with mechanical polarization controllers were used for ensuring TM polarized light at the input of the SOI-DLSPP chip. A SRS DG535 digital delay/pulse generator operating at 20 KHz controlled dynamically the switching state of the MZI based on the TO effect. For the BER measurement performance, the electrical pulsed control signal was replaced by a DC current. The multi-channel signal that was received at the MZI's output was amplified by a two stage EDFA (EDFA2 with –15 dBm output power for an input power of −22 dBm and EDFA3) that included a 5 nm OBPF between the two stages for noise rejection. The WDM signal was demultiplexed into its constituent wavelengths through a narrow OBPF (0.8 nm). Each data channel was then split and launched concurrently into a 30 GHz HP 83480 A sampling oscilloscope and a 10 GHz 3-dB bandwidth DSC-R402PIN photo-receiver attached to a 10 Gb/s MP1764C Anritsu error detector. The 10 GHz reference signal for the transmitter and the receiver was provided by a 20 GHz MG3692B Anritsu signal generator.

### BER measurements

The BER curves were obtained by measuring the BER values for increasing mean optical signal power levels launched at the photo-receiver and applying a fitting curve to the measured points, following the method described in 38. The B2B measurements were performed by replacing the A-MZI with a straight 3 mm long silicon waveguide section that had 27.3 dB total fiber-to-fiber losses originating from 26 dB losses due to the two TM grating couplers and 4.4 dB/cm silicon propagation losses.

## Author Contributions

S.P., D.K., K.V., D.A., H.A. and N.P. devised the experimental setup and carried out the experimental testing procedure and measurements. J.-C.W., K.H., L.M. and A.D. designed the widened plasmonic MZI arm for providing the phase offset and fabricated the plasmonic parts of the A-MZI structure. M.B. fabricated the SOI motherboard. T.T. designed the silicon parts and prepared the mask layout. A.K. and S.B. characterized the sample and performed the wire-bonding. S.P., D.K., K.V. and N.P. analyzed the results and prepared the figures. All authors co-wrote the paper.

## Supplementary Material

Supplementary Information Supplementary Fig. S1

## Figures and Tables

**Table 1:** Comparison with other TO SOI- and polymer-based nanophotonic MZI switches

Reference work	Active waveguide technology	Phase arm length (active region in *μ*m)	Power consumption (P in mW)	Switching time (τ in *μ*s)	Power-time product (P×τ in mW·*μ*s)
22	SOI	200	20	2.8	56
23	SOI	1000	0.49	144	70.56
24	SOI	100	0.54	141	76.14
25	SOI	700	50	3.5	175
26	SOI	6300	6.5	14	91
27	Polymer	300	1.85	700	1295
28	Polymer	100	4	200	800
Current work	PMMA-loaded SPP	60	13.1	3.8	49.78
