# Supplementary material for: Active plasmonics in WDM traffic switching applications
Source: Sci Rep. 2012 Sep 12;2:652. doi: 10.1038/srep00652 (PMC3439651; doi:10.1038/srep00652)
Supplement: Supplementary Information — Supplementary Fig. S1 [file srep00652-s1.pdf]

Supplementary Information

"Active plasmonics in WDM traffic switching applications"

Sotirios Papaioannou[1,2,*], Dimitrios Kalavrouziotis[3], Konstantinos Vyrsokinos[2], Jean-Claude Weeber[4], Karim Hassan[4], Laurent Markey[4], Alain Dereux[4], Ashwani Kumar[5], Sergey I. Bozhevolnyi[5], Matthias Baus[6], Tolga Tekin[7], Dimitrios Apostolopoulos[3], Hercules Avramopoulos[3] & Nikos Pleros[1,2]

[1]Department of Informatics, Aristotle University of Thessaloniki, 54124 Thessaloniki, Greece, [2]Informatics and Telematics Institute, Center for Research and Technology Hellas, 57001 Thessaloniki, Greece, [3]School of Electrical and Computer Engineering, National Technical University of Athens, 15780 Zografou, Athens, Greece, [4]Institut Carnot de Bourgogne, University of Burgundy, F-21078 Dijon Cedex, France, [5]Faculty of Engineering, Institute of Technology and Innovation, University of Southern Denmark, DK-5230 Odense M, Denmark, [6]AMO GmbH, 52074 Aachen, Germany, [7]Fraunhofer IZM, D-13355 Berlin, Germany.

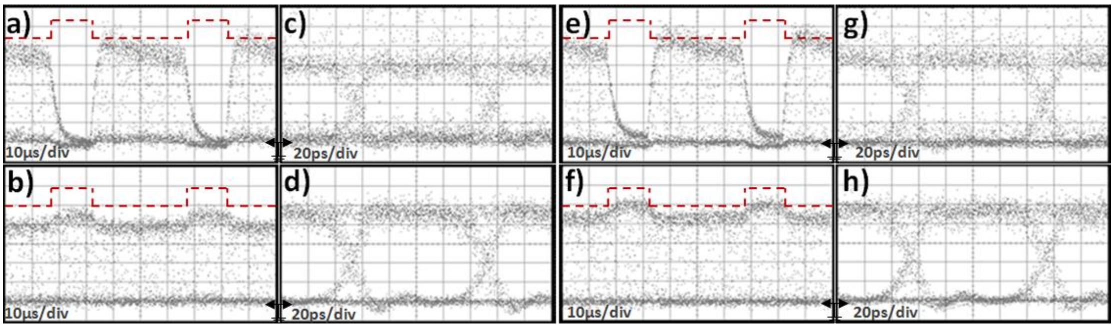

Supplementary Fig. S1: Data injection. Modulation with 15μs electrical rectangular pulses at 20KHz repetition rate for 10Gb/s (a) data trace at the CROSS port (channel 3), (b) data trace at the BAR port (channel 3), (c) eye diagram at the CROSS port (channel 3), (d) eye diagram at the BAR port (channel 3), (e) data trace at the CROSS port (channel 4), (f) data trace at the BAR port (channel 4), (g) eye diagram at the CROSS port (channel 4), (h) eye diagram at the BAR port (channel 4).
